# Investigation of the Role of Pituitary Adenylate Cyclase-Activating Peptide (PACAP) and Its Type 1 (PAC1) Receptor in Uterine Contractility during Endometritis in Pigs

**DOI:** 10.3390/ijms23105467

**Published:** 2022-05-13

**Authors:** Barbara Jana, Jarosław Całka, Krzysztof Witek

**Affiliations:** 1 Division of Reproductive Biology, Institute of Animal Reproduction and Food Research of the Polish Academy of Sciences, Tuwima 10, 10-078 Olsztyn, Poland; k.witek@pan.olsztyn.pl; 2 Department of Clinical Physiology, Faculty of Veterinary Medicine, University of Warmia and Mazury, Oczapowskiego 13, 10-718 Olsztyn, Poland; calkaj@uwm.edu.pl

**Keywords:** endometritis, myometrium, PAC1 receptor expression, uterine contractility, pig

## Abstract

Uterine inflammation is a common pathology in animals, leading to disturbances in reproductive processes and reduced production profitability. Pituitary adenylate cyclase-activating peptide (PACAP) effects at the uterine level during inflammation are not known. In the current study, we analyzed the relative PACAP type 1 receptor (PAC1R) mRNA transcript and protein abundances in the myometrium (MYO), as well s PACAP and PAC1R involvement in the contractile function of inflamed pig uterus. To that end, *E. coli* suspension (*E. coli* group) or saline (SAL group) was injected into the uterine horns or laparotomy was performed (CON group). Eight days after the bacteria injections, severe acute endometritis and a reduced relative abundance of PAC1R protein in the MYO were observed. Compared to the period before PACAP in vitro administration, PACAP (10^−7^ M) in the CON and SAL groups decreased in amplitude in the MYO and endometrium (ENDO)/MYO, whereas in the *E. coli* group, increased amplitude in the MYO and reduced amplitude in the ENDO/MYO were observed. In the *E. coli* group, PACAP enhanced the amplitude in the MYO (10^−7^ M) and decreased the amplitude in the ENDO/MYO (10^−8^ M) compared with other groups. PACAP (10^−7^ M) increased the frequency of both kinds of strips in the CON and SAL groups compared with the pretreatment period. PACAP (both doses) did not significantly change the frequency in the *E. coli* group, whereas in response to PACAP (10^−7^ M), the frequency was reduced compared to other groups. In the MYO, PAC1R antagonist decreased the amplitude reduction (CON and SAL groups) and reversed a rise in PACAP (10^−7^ M)-evoked amplitude (*E. coli* group). PAC1R blocking reversed (MYO) and abolished (ENDO/MYO) the stimulatory effect of PACAP (10^−7^ M) on the frequency (CON and SAL groups). PAC1R antagonist and PACAP (10^−7^ M) evoked the appearance of frequency depression in both kinds of strips (*E. coli* group). In summary, in pigs, severe acute endometritis reduces the relative abundance of PAC1R protein in the MYO, and PAC1R mediates the influence of PACAP on inflamed uterus contractility.

## 1. Introduction

Endometritis and metritis are common and serious diseases in animals that result in disturbances in reproductive processes and reduced production profitability [1,2]. Bacterial organisms are mainly responsible for the formation of uterine inflammations, whereas difficult parturition and retention of the fetal membranes are primarily situations that favor the development of these pathologies [3]. For the origin, development and maintenance of endometritis and metritis, dysfunction of the uterine immune system and/or contractility are of great importance [4,5]. In severe conditions of inflammation associated with loss or reduced contractile activity of the uterus, accumulation of inflammatory exudate in the uterine cavity and disorders of reproductive processes occur [6,7]. With regard to the control of inflamed uterus contractility, the importance of metabolites of arachidonic acids, such as prostaglandin (PG)F2α [8], PGE2 [9], PGI2 [10], leukotriene (LT)C4 and LTD4 [11] in pigs, as well as PGF2α during the postpartum period in cows [12], have been reported. Studies have also been devoted to the role of noradrenaline (NA) [9,10,13,14,15], acetylcholine (ACh) [16,17], neuropeptide Y (NPY) [18], somatostatin [19], vasoactive intestinal peptide (VIP) [20] and galanin (GAL) [21] in the contractility of porcine inflamed uterus, together with the function of receptors for these neurotransmitters.

Pituitary adenylate cyclase-activating peptide (PACAP), appearing as a 27- or 38-aminoacid neuropeptide, belongs to the VIP/secretin/glucagon peptide family. PACAP acts by binding to different G-protein-coupled receptors, including PACAP type 1 (PAC1) receptor, VIP subtype 1 (VPAC1) and VIP subtype 2 (VPAC2) receptors. PAC1 receptor is the specific receptor that selectively binds PACAP-27 and PACAP-38, whereas VPAC1 and VPAC2 receptors bind VIP and PACAP with equal affinity [22]. PACAP and its receptors are expressed in the central nervous system and in the peripheral organs, such as the endocrine, genitourinary, respiratory and cardiovascular systems and gastrointestinal tract, where they are involved in many biological functions under physiological and pathological conditions. This peptide acts mainly as a neurotransmitter and/or neuromodulator and exerts neurotrophic and neuroprotective influences [23,24].

It is known that under physiological conditions, PACAP immunoreactivity is present in the uterus-innervating neurons of the pig dorsal root ganglia (DRGs) [25], as well as in rat lumbosacral DRGs [26] and in the nerve fibers of the pig paracervical ganglion (PCG) [27]. PACAP-ergic nerve fibers occur both in the endometrial and myometrial layers in women [28], rats [26,29] and pigs [30]. PACAP plays an important role in the control of reproductive function in females, including relaxation of the human non-pregnant myometrium [28] and the rabbit ovary artery and oviduct smooth muscle [31]. PACAP is also important for human trophoblast cell functions [32] and early mouse embryo development [33]. With regard to PACAP-ergic innervation of the inflamed uterus, it has been reported that endometritis increased uterine perikarya populations expressing PACAP with or without substance P content in the DRG Th10-S4 of pigs [25].

In light of the above data, it was hypothesized that endometritis influences PAC1 receptor patterns in myometrium and that this receptor participates in the PACAP action on contractility of inflamed uteri. Improved knowledge of the changes and importance of PAC1 receptor in uterine inflammatory contractility will result in enhanced data on the receptor-based neurogenic control of uterine inflammation, which will markedly improve the results of prophylaxis and treatment of uterine inflammation in animals. It is also possible that findings from research on the domestic pig model (with a high similarity of anatomical structures and physiological processes to those of humans) will allow for better recognition of uterine inflammation mechanisms in women [34]. The present work was therefore undertaken to determine the action of endometritis in gilts on (1) the relative PAC1 receptor mRNA transcript and protein abundances in myometrium, as well as (2) the contribution of PAC1 receptor in PACAP-evoked uterine amplitude and frequency of contraction.

## 2. Results

### 2.1. Relative PAC1 Receptor mRNA Transcript Abundance

**Figure 1:**
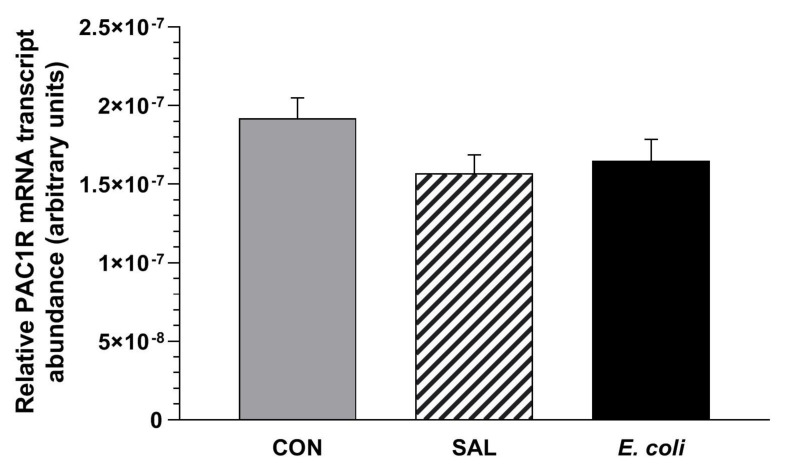
Relative pituitary adenylate cyclase-activating peptide receptor (PAC1R) mRNA transcript abundances in the myometrial layer of gilts from the control (CON), saline (SAL) and *E. coli* (*E. coli*) groups, estimated by real-time PCR. Relative PAC1R mRNA transcript abundances are expressed as the mean ± SEM of ratios in relation to glyceraldehyde-3-phosphate dehydrogenase (GAPDH).

The relative PAC1 receptor mRNA transcript abundances in the myometrium did not differ significantly in the CON, SAL and Escherichia coli (*E. coli*) groups (Figure 1).

### 2.2. Relative PAC1 Receptor Protein Abundance

Duodenal proteins (mouse and porcine) utilized as the positive controls showed bands at 68 kDa, and they were assumed to be PAC1 receptor proteins (Supplementary Figure S1). The band was not observed following omission of the primary antibody (data not shown). Western blot analysis of gilt myometrial layers showed protein bands at 68 kDa for the PAC1 receptor (Supplementary Figure S2).

**Figure 2:**
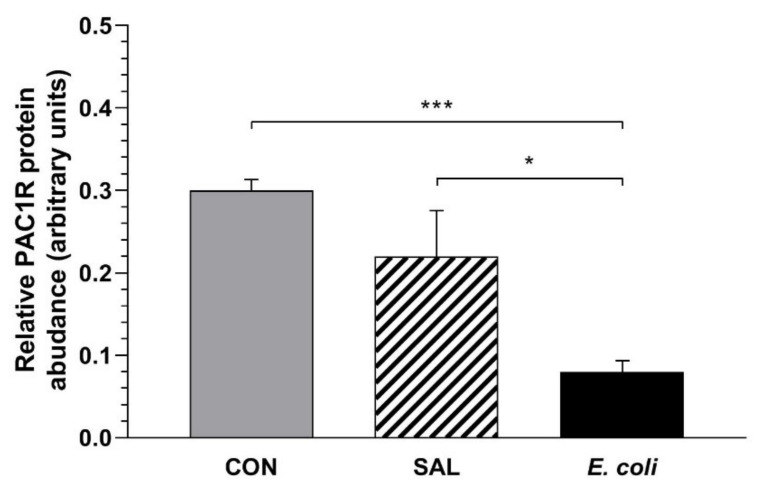
Relative pituitary adenylate cyclase-activating peptide receptor (PAC1R) protein abundances in the myometrial layer of gilts from the control (CON), saline (SAL) and *E. coli* (*E. coli*) groups, estimated by Western blot analysis. The relative PAC1R protein abundances are expressed as the mean ± SEM of ratios in relation to glyceraldehyde-3-phosphate dehydrogenase (GAPDH). The blot with representative bands for each group is presented in Supplementary Figure S2. * *p* < 0.05, *** *p* < 0.001 compared between groups.

In the myometria of the *E. coli* group, the relative PAC1 receptor protein abundance was reduced compared to the CON (*p* < 0.001) and SAL (*p* < 0.05) groups (Figure 2).

### 2.3. Localization of PAC1 Receptor

**Figure 3:**
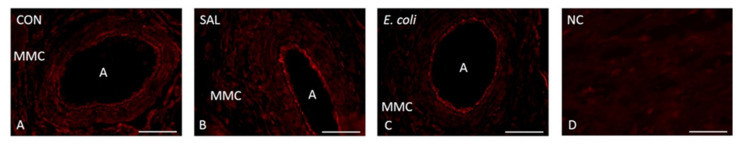
Representative pictures show pituitary adenylate cyclase-activating peptide receptor (PAC1R) immunostaining in the myometrial layer of gilts from the control (CON), saline (SAL) and *E. coli* (*E. coli*) groups. Positive immunoreaction to PAC1R is visible in muscle cells and arteries (endothelium, muscle layer) of the myometrium of the control (**A**), saline-injected (**B**) and inflamed (**C**) uteri. Negative control (NC) for PAC1R (**D**) was obtained by omitting the primary antibody. MMC—myometrial muscle cells; A—artery. The scale bar of each image is 50 µm in length.

Immunofluorescent staining of the pig duodenum as a positive control revealed the presence of a PAC1 receptor (Supplementary Figure S3). This receptor was not found after omission of the primary antibody (Figure 3D). PAC1 receptor immunoreactivity was visible in the muscle cells and blood vessels (endothelium and muscle layer) of myometria in the CON (Figure 3A), SAL (Figure 3B) and *E. coli* (Figure 3C) groups.

### 2.4. Influence of PACAP Alone or Combined with PAC1 Receptor Antagonist on the Contractile Amplitude of the Myometrium

#### 2.4.1. Comparison of PACAP Influence in the Particular Groups with That in the Period before Its Application

**Figure 4:**
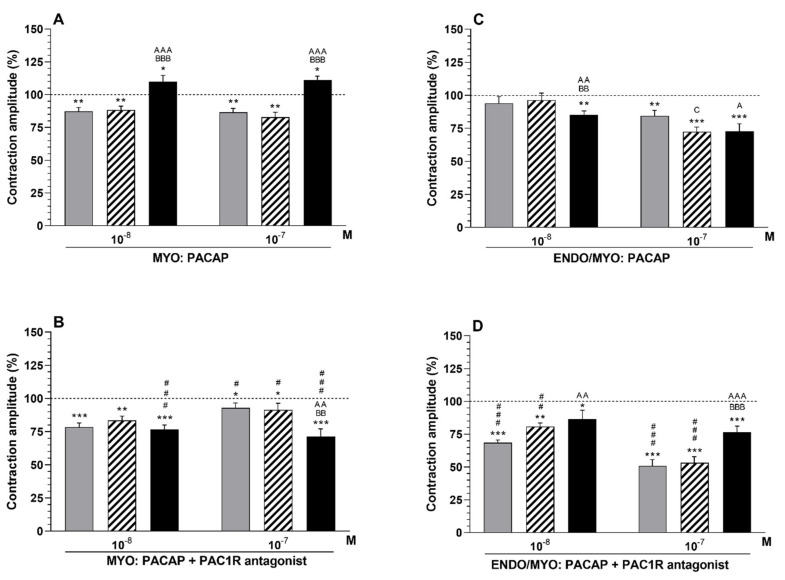
Influence of pituitary adenylate cyclase-activating peptide (PACAP) alone (**A**,**C**) and PACAP receptor (PAC1R) antagonist with PACAP (**B**,**D**) on the contractile amplitude in the myometrium (**A**,**B**) and endometrium/myometrium (**C**,**D**) strips of gilts from the CON (grey bars), SAL (hatched bars) and *E. coli* (black bars) groups. Results were calculated for five gilts in each group. The actions of the antagonist (a dose of 10*^−^*^6^ M) and particular PACAP doses are depicted as percentage (mean ± SEM) changes from the basal (pre-treatment period) amplitude taken as 100% (horizontal lines). * *p* < 0.05, ** *p* < 0.01, *** *p* < 0.001 compared to the basal value in each group; ^A^ *p* < 0.05, ^AA^ *p*<0.01, ^AAA^ *p*<0.001 between the CON and *E. coli* groups for the same treatment; ^BB^ *p* < 0.01, ^BBB^ *p* < 0.001 between the SAL and *E. coli* groups for the same treatment; ^C^ *p* < 0.05 between the CON and SAL groups for the same treatment; ^#^ *p*<0.05, ^##^ *p* < 0.01, ^###^ *p* < 0.001 between the antagonist with PACAP action versus PACAP action alone for the same group/tissue/PACAP dose.

The amplitude in myometria after using PACAP (10*^−^*^8^, 10*^−^*^7^ M) was reduced (*p* < 0.01) in the CON and SAL groups, whereas it was enhanced (*p* < 0.05) in the *E. coli* group (Figure 4A).

#### 2.4.2. Comparison of PACAP Influence between Groups

The myometrial amplitude in the *E. coli* group was enhanced (*p* < 0.001) by PACAP (10*^−^*^8^, 10*^−^*^7^ M) compared to the CON and SAL groups (Figure 4A).

#### 2.4.3. Comparison of the Influence of PAC1 Receptor Antagonist and PACAP in the Particular Groups with That in Period before Their Application

Use of PAC1 receptor antagonist (10*^−^*^6^ M) and PACAP decreased the amplitude in the myometria of the CON (PACAP—10*^−^*^8^ M: *p* < 0.001, 10*^−^*^7^ M: *p* < 0.05), SAL (PACAP—10*^−^*^8^ M: *p* < 0.01, 10*^−^*^7^ M: *p* < 0.05) and *E. coli* (PACAP—10*^−^*^8^, 10*^−^*^7^ M: *p* < 0.001) groups (Figure 4B).

#### 2.4.4. Comparison of the Influence of PAC1 Receptor Antagonist and PACAP between Groups

The amplitude in the myometria of the *E. coli* group was lower (*p* < 0.01) than in the CON and SAL groups in response to PAC1 receptor antagonist (10*^−^*^6^ M) and PACAP (10*^−^*^7^ M) (Figure 4B).

#### 2.4.5. Comparison of the Influence of PAC1 Receptor Antagonist and PACAP with That of PACAP Alone

After using the antagonist (10*^−^*^6^ M) and PACAP (10*^−^*^8^, 10*^−^*^7^ M), the amplitude in the myometrium of the *E. coli* group was lower (*p* < 0.001) compared to following the application of PACAP alone (Figure 4A,B). A similar result (*p* < 0.05) was noted in the myometria of the CON and SAL groups in response to the antagonist and PACAP (10*^−^*^7^ M) versus PACAP action.

### 2.5. Influence of PACAP Alone or Combined with PAC1 Receptor Antagonist on the Contractile Amplitude of the Endometrium/Myometrium

#### 2.5.1. Comparison of the Influence of PACAP in the Particular Groups with That in the Period before Its Application

PACAP (10*^−^*^7^ M) decreased the amplitude in the endometria/myometria of the CON (*p* < 0.01) and SAL (*p* < 0.001) groups. A reduction in this parameter was also noted in the *E. coli* group after using PACAP (10*^−^*^8^ M—*p*<0.01, 10*^−^*^7^ M—*p* < 0.001) (Figure 4C).

#### 2.5.2. Comparison of the Influence of PACAP between Groups

In response to PACAP (10*^−^*^8^ M), the amplitude in the endometria/myometria of the *E. coli* group was lower (*p* < 0.01) than in the CON and SAL groups (Figure 4C). In response to PACAP (10*^−^*^7^ M), this parameter was decreased (*p* < 0.05) in the SAL and *E. coli* groups in relation to the CON group.

#### 2.5.3. Comparison of the Influence of the PAC1 Receptor Antagonist and PACAP in the Particular Groups to That in the Period before Their Application

PAC1 receptor antagonist (10^−6^ M) with PACAP decreased the amplitude in endometria/myometria of the CON (PACAP—10^−8^, 10^−7^ M: *p* < 0.001), SAL (PACAP—10^−8^ M: *p* < 0.01, 10^−7^ M: *p* < 0.001) and *E. coli* (PACAP—10^−8^ M: *p* < 0.05, 10^−7^ M: *p* < 0.001) groups (Figure 4D).

#### 2.5.4. Comparison of the Influence of the PAC1 Receptor Antagonist and PACAP between Groups

The application of PAC1 receptor antagonist (10*^−^*^6^ M) with PACAP (10*^−^*^7^ M) led to a rise (*p* < 0.001) in the amplitude in endometria/myometria of the *E. coli* group compared to the other groups (Figure 4D). A similar result (*p* < 0.01) was observed between the *E. coli* and CON groups in response to the antagonist and PACAP at a dose of 10*^−^*^8^ M.

#### 2.5.5. Comparison of the Influence of the PAC1 Receptor Antagonist and PACAP with That of PACAP Alone

In response to the antagonist (10*^−^*^6^ M) and PACAP, the amplitude in the endometrium/myometrium of the CON (PACAP—10*^−^*^8^, 10*^−^*^7^ M: *p* < 0.001) and SAL (PACAP—10*^−^*^8^ M: *p* < 0.01, 10*^−^*^7^ M: *p* < 0.001) groups was lower compared to that of PACAP action alone (Figure 4C,D).

### 2.6. PACAP Influence Alone or Combined with PAC1 Receptor Antagonist on the Contractile Frequency of the Myometrium

#### 2.6.1. Comparison of the Influence of PACAP in the Particular Groups with That of the Period before Its Application

**Figure 5:**
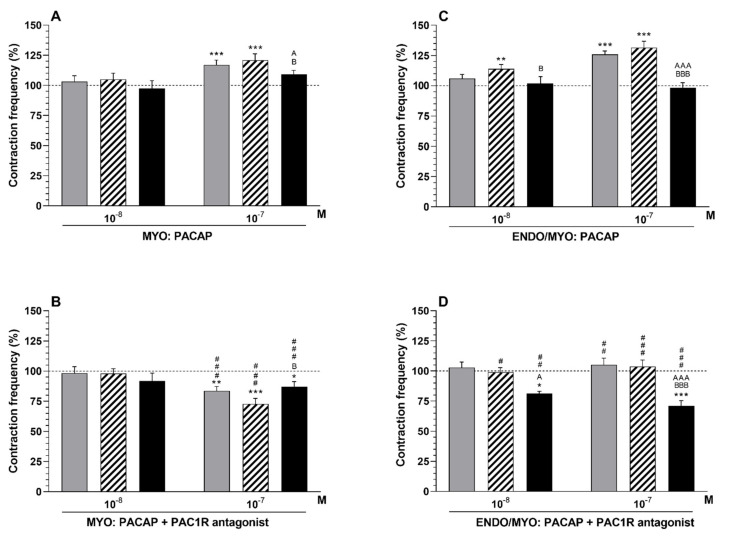
Influence of pituitary adenylate cyclase-activating peptide (PACAP) alone (**A**,**C**) and PACAP receptor (PAC1R) antagonist with PACAP (**B**,**D**) on the contractile frequency in the myometria (**A**,**B**) and endometrium/myometrium (**C**,**D**) strips of gilts from the CON (grey bars), SAL (hatched bars) and *E. coli* (black bars) groups. Results were calculated for five gilts in each group. The actions of the antagonist (a dose of 10*^−^*^6^ M) and particular PACAP doses are depicted as the percentage (mean ± SEM) change from the basal (pre-treatment period) frequency, taken as 100% (horizontal lines). * *p* < 0.05, ** *p* < 0.01, *** *p* < 0.001 compared to the basal value in each group; ^A^ *p* < 0.05, ^AAA^ *p* < 0.001 compared between the CON and *E. coli* groups for the same treatment; ^B^ *p* < 0.05, ^BBB^ *p* < 0.001 compared between the SAL and *E. coli* groups for the same treatment; ^#^ *p* < 0.05, ^##^ *p* < 0.01, ^###^ *p* < 0.001 compared between the antagonist with PACAP action versus PACAP action alone for the same group/tissue/PACAP dose.

PACAP (10*^−^*^7^ M) increased (*p* < 0.001) the frequency in the CON and SAL groups (Figure 5A).

#### 2.6.2. Comparison of PACAP Influence between Groups

In the *E. coli* group, the myometrial frequency was lower (*p* < 0.05) in response to PACAP (10*^−^*^7^ M) than in other groups (Figure 5A).

#### 2.6.3. Comparison of the Influence of PAC1 Receptor Antagonist and PACAP in the Particular Groups with That in the Period before Their Application

The myometrial frequency in the CON (*p* < 0.01), SAL (*p* < 0.001) and E. coli (*p* < 0.05) groups was decreased by the antagonist (10^−6^ M) and PACAP (10^−7^ M) (Figure 5B).

#### 2.6.4. Comparison of the Influence of PAC1 Receptor Antagonist and PACAP between Groups

After application of the antagonist (10*^−^*^6^ M) with PACAP (10*^−^*^7^ M), the frequency in the myometria of the *E. coli* group was enhanced (*p* < 0.05) in relation to that in the SAL group (Figure 5B).

#### 2.6.5. Comparison of the Influence of PAC1 Receptor Antagonist and PACAP with That of PACAP Alone

The antagonist (10*^−^*^6^ M) and PACAP (10*^−^*^7^ M) led to a reduction (*p* < 0.001) in the myometrial frequency of the CON, SAL and *E. coli* groups compared PACAP alone (Figure 5A,B).

### 2.7. Influence of PACAP Alone or Combined with PAC1 Receptor Antagonist on the Contractile Frequency of the Endometrium/Myometrium

#### 2.7.1. Comparison of the Influence of PACAP in the Particular Groups with That in the Period before Its Application

PACAP increased the frequency in the endometria/myometria of the CON (PACAP—10*^−^*^7^ M: *p* < 0.001) and SAL (PACAP—10*^−^*^8^ M: *p* < 0.01, 10*^−^*^7^ M: *p* < 0.001) groups (Figure 5C).

#### 2.7.2. Comparison of the Influence of PACAP between Groups

The frequency in the *E. coli* group after using PACAP (10*^−^*^7^ M) was reduced (*p* < 0.001) compared to other groups (Figure 5C). This situation (*p* < 0.05) was also observed between the *E. coli* and SAL groups in response to PACAP at a dose of 10*^−^*^8^ M.

#### 2.7.3. Comparison of the Influence of PAC1 Receptor Antagonist and PACAP in the Particular Groups with That in the Period before Their Application

In the *E. coli* group, the PAC1 receptor antagonist (10*^−^*^6^ M) with PACAP reduced (PACAP—10*^−^*^8^ M: *p* < 0.05, 10*^−^*^7^ M: *p* < 0.001) the frequency in the endometrium/myometrium (Figure 5D).

#### 2.7.4. Comparison of the Influence of PAC1 Receptor Antagonist and PACAP between Groups

After using the PAC1 receptor antagonist (10*^−^*^6^ M) and PACAP (10*^−^*^7^ M), the frequency in the endometria/myometria of the *E. coli* group was lower (*p* < 0.001) than in other groups (Figure 5D). The antagonist and PACAP (10*^−^*^8^ M) also decreased (*p* < 0.05) this parameter in the *E. coli* group in relation to the CON group.

#### 2.7.5. Comparison of the Influence of PAC1 Receptor Antagonist and PACAP with That of PACAP Alone

In response to the antagonist (10*^−^*^6^ M) and PACAP, the frequency in the endometria/myometria of the CON (PACAP—10*^−^*^7^ M: *p* < 0.01), SAL (PACAP—10*^−^*^8^ M: *p* < 0.05, 10*^−^*^7^ M: *p* < 0.001) and *E. coli* (PACAP—10*^−^*^8^ M: *p* < 0.01, 10*^−^*^7^ M: *p* < 0.001) groups was reduced compared to PACAP action alone (Figure 5C,D).

## 3. Discussion

Among many factors influencing the origin, development and maintenance of uterine inflammation, contractility is extremely important. It is therefore warranted to further understand the neural regulation of uterine contraction in inflammation. In connection with this, in the current study, we determined the relative PAC1 receptor mRNA transcript and protein abundances in the myometrium, as well as the contribution of the PACAP/PAC1 receptor system, in the contractility of pig uteri with severe acute endometritis. The macroscopic and histopathological assessments of uteri used in the current study were published earlier [13].

To date, in reference to female reproductive organs, the presence of PAC1 receptor has been demonstrated only in rat ovarian granulosa [35] and corpora lutea [36] cells, as well as in human [37] and rat [37,38] placenta. Thus, the current study (for the first time) shows the relative PAC1 receptor mRNA transcript and protein abundances in uterine tissues under physiological and inflammatory conditions. Although endometritis did not significantly affect the relative PAC1 receptor mRNA transcript abundance in the myometrium (present study), this pathology reduced the relative PAC1 receptor protein abundance compared to the CON and SAL groups. A drop in PAC1 receptor expression was also noted in rat urothelium and destructor smooth muscle after acute chemically-induced cystitis in rats, whereas it was increased during intermediate and chronic inflammation [39]. An enhancement in PAC1 receptor expression was revealed in the murine inflamed lung [40]. The relative PAC1 receptor protein abundances in the myometria of the CON and SAL groups were not statistically different, suggesting that saline uterine administration had no significant effect on the relative protein abundance of this receptor. Moreover, the current study confirmed the PAC1 receptor in the muscle and blood vessel cells in the myometria of all studied groups, indicating that these cells bind PACAP. It is possible that PACAP regulates the function of myometrial blood vessels and muscle cells under physiological and inflammatory conditions in pigs through this receptor. In reference to blood vessels, it is known, for example, that PACAP and PAC1 receptors are expressed in the rat placenta, including chorionic vessels [38], and that PACAP inhibits the contractile effect of NA on the intramyometrial arteries of pregnant [41] and non-pregnant [28] women. The PACAP/PAC1 receptor system is also important for the maintenance of human corneal endothelial integrity [42] and normal pulmonary vascular tone [43], as well as for the function of extracerebral vessels in migraine patients [44]. It is worth noting that PACAP plays a role in the release of PGs (F2α, E2 and I2), LTC4 and thromboxane A1 from myometria of gilts, such as those used in the present study (B. Jana et. al., unpublished data) through the PAC1 receptor.

In the contractile investigations of the uteri described in the current study, ACh was used to assess the viability and serviceability of strips for study. After stimulation with ACh, an increase in the frequency in the CON, SAL and *E. coli* groups was observed, whereas the amplitude increased in the CON and SAL groups and decreased in the *E. coli* group [16], which is in agreement with previous works [9,10].

As mentioned above, the literature data on the role of PACAP in the contractile activity of the reproductive tract are very limited. The current study is the first to investigate the effect of PACAP on the contractility of the healthy uterus in pigs, as well as on the inflamed uterus. Compared to the period before PACAP application, this peptide decreased the amplitude and increased the frequency in the myometria and endometrium/myometrium strips of the CON and SAL groups. A drop in the amplitude under the influence of PACAP is in line with studies of the human uterus [28] and the rabbit ovarian artery and smooth muscle of the oviduct [31]. Moreover, existing data show the relaxatory action of PACAP on the contractile function of the gastrointestinal tract, for example, the stomach, ileum and colon [45,46,47,48], as well as the intravesical ureter [49]. Based on the changes in the values of the studied contractile parameters in response to PAC1 receptor antagonist and PACAP compared to the PACAP effect alone, the current study revealed that the PAC1 receptor participates in the PACAP contractility of the healthy pig uterus. Moreover, no noticeable differences were observed in the relative PAC1 receptor mRNA transcript and protein abundances in the myometrium between the CON and SAL groups.

Compared to the period before PACAP treatment, in the *E. coli* group, PACAP increased the amplitude in the myometrium and decreased the amplitude in the endometrium/myometrium and did not significantly alter the frequency in either kind of strip. Amplitude changes in the inflamed uterus after the use of PAC1 receptor antagonist with PACAP (reversal of the myometrial stimulating effect) in relation to the PACAP action alone indicate that the PACAP action on amplitude is mediated by the PAC1 receptor. In turn, a drop in the frequency in the inflamed uterus observed in response to the antagonist and PACAP indicates the importance of PACAP in maintaining uterine frequency during inflammation. The role of the PACAP/PAC1 receptor system in the regulation of smooth muscle destructor motility was previously observed in chemically-evoked cystitis in rats [50]. Moreover, after *E. coli* treatment, the amplitude in the myometrium was higher, whereas the amplitude in the endometrium/myometrium and the frequency (both kinds of strips) was lower than in the CON and SAL groups. The rise in the myometrial amplitude in the *E. coli* group could be due to the reduced relative PAC1 receptor protein abundance in the myometrium, as shown in the present study. In contrast, the decrease in endometrial/myometrial amplitude in this group may be due to the indirect influence of PACAP. Previous studies have demonstrated the role of PACAP in the release of PGE2, nitric oxide (NO), ACh and catecholamines from different tissues [51,52,53] and the interplay between PACAP and NO in the contractility of the mouse ileum [54]. It was also reported that the PGE2 [55] and NO [56] synthesis was increased in the pig endometrium during inflammation and that PGE2 [9], ACh [16] and NA [10] are able to lower the contraction amplitude in the inflamed porcine uterus.

In addition to the substances listed above, the drop in the amplitude in the porcine uterus with endometritis is caused by NPY, VIP and GAL [18,20,21], whereas PGF2α, PGI2, LTC4 and LTD4 increased the value of this parameter [8,9,11]. In the present report, the authors extended those findings by demonstrating that PACAP increases the myometrial amplitude, which may lead to the removal of inflammatory exudate from the uterine lumen. In addition, the role of the PAC1 receptor in increasing the amplitude of the inflamed uterus by PACAP observed in the current study can be exploited to develop drugs that raise uterine contractility under inflammatory conditions. As a result, such improvements in the effectiveness of treatment and prevention of postpartum diseases of the reproductive system will translate into better reproductive indicators and increased profitability of animal production.

## 4. Materials and Methods

### 4.1. Animals and Research Procedures

The research was performed on gilts (Large White × Landrace, age 7–8 months, body weight/BW/90–120 kg, the “Wronie” breeding farm, Wronie, Poland) in which reproductive disturbances were not observed. A tester boar was used to determine behavioral estrus. Three days before the start of the study, the gilts were transported from a farm to the animal house (University of Warmia and Mazury, Olsztyn, Poland), where they were kept in individual pens (an area of about 5 m^2^) under 14.5 ± 1.5 h of natural daylight and 9.5 ± 1.5 h of night and a temperature of 18 ± 2 °C. The gilts were fed a commercial diet and had access to water ad libitum. All research procedures were carried out taking into account the relevant Polish and EU regulations in the field of animal protection and welfare (Regulation 26/2014 implementing the EU Directive 2010/63/EU) and were approved by the Local Ethics Committee (Consent No. 65/2015).

The pigs were divided (randomly) into the following groups on day 3 of the second estrous cycle (day 0 of the study): *E. coli* (*E. coli*), saline (SAL) and control (CON) (five pigs in each group). The details of experimental procedures were provided by Jana et al. [10]. Atropine (0.05 mg/kg BW; Atropinum sulf. WZF, Warszawskie Zakłady Farmaceutyczne Polfa S.A., Poland), azaperone (2 mg/kg BW; Stresnil, Janssen Pharmaceutica, Beerse, Belgium) and ketamine hydrochloride (10 mg/kg BW; Ketamina, Biowet, Puławy, Poland) were used to premedicate the animals. Ketamine hydrochloride (supplementary doses: 1 mg/kg BW every 5 min) was also administered to induce and maintain general anesthesia. Median laparotomy was performed, and in the *E. coli* group, 50 mL of *E. coli* suspension (strain O25:K23/α/:H1; Department of Microbiology, National Veterinary Research Institute, Puławy, Poland) containing 10^9^ colony-forming units/mL was injected into each uterine horn, whereas in the SAL group, 50 mL of saline solution was injected. In the pigs of the CON group, only median laparotomy was performed. All pigs were left untreated during the period from surgery to euthanasia. On day 8 of the experiment (the expected day 11 of the estrous cycle), the uteri were collected following euthanasia (overdose of the ketamine hydrochloride). Fragments of the horn from the paraoviductal, middle and paracervical parts were collected for analysis. For real-time reverse transcriptase-polymerase chain reaction (real-time RT-PCR) and Western blot analyses, the endometrial and myometrial layers were divided using a scalpel blade and a dissecting microscope, and fragments of the myometrium about the thickness of the entire layer were snap-frozen in liquid nitrogen and stored at −80 °C. In turn, for immunofluorescence study, the uterine tissues were fixed in a 4% paraformaldehyde solution (pH 7.4) for 24 h. They were then rinsed in 0.1 M phosphate-buffered saline (PBS, pH 7.4) and cryoprotected in an 18% buffered solution of sucrose (pH 7.4) until sectioning. Fragments of the horn of the uterus from its middle part were transported on ice to the laboratory (within 5 min following collection) to measure contractility.

### 4.2. RNA Extraction and Real-Time RT-PCR

To isolate total RNA from myometrial tissues, they were homogenized in TRI reagent solution (Invitrogen, Thermo Fisher Scientific, Waltham, MA, USA) using a FastPrep 24 homogenizer (MP Biomedicals, LCC, Irvine, CA, USA). For phase separation, a BCP reagent (Molecular Research Center Inc., Cincinnati, OH, USA) was used, and the RNA was then purified using an RNeasy mini kit (QIAGEN, Valencia, CA, USA) according to the manufacturer’s instructions. Until further use, RNA was stored at −80 °C in RNase-free water with the addition of RNAse inhibitor (Applied Biosystems, Thermo Fisher Scientific, USA). The quality and quantity of extracted RNA were determined using a NanoDrop 1000 (Thermo Fisher Scientific, USA) and an Agilent 2100 bioanalyzer (Agilent Technologies, USA). RNA with an RNA integrity number in the range 7.0–9.6 was used in real-time RT-PCR.

Real-time RT-PCR was carried out using TaqMan tests (Table 1) and a one-step PCR master mix (Applied Biosystems, Waltham, MA, USA). Each reaction (10 μL) contained 15 ng of total RNA in a volume of 3 µL, 5 μL 2 × TaqMan RT-PCR mix, 0.25 μL 40 × TaqMan RT enzyme mix, 0.5 μL 20 × TaqMan gene expression assay and 1.25 μL RNase-free water (Applied Biosystems). The RT-PCR reaction was performed in duplicates in 384-well plates under the following conditions: reverse transcription for 15 min at 48 °C, initial denaturation (for 10 min) at 95 °C, followed by 45 cycles of 15 s of denaturation at 95 °C and 1 min of annealing at 60 °C in an ABI Prism 7900HT system (Applied Biosystems). The negative control was prepared by replacing the RNA template with RNase-free water. Obtained data were analyzed using the Miner method [57]. The NormFinder algorithm was utilized to choose the most stable housekeeping gene among β-actin (ACTB), hypoxanthine guanine phosphoribosyl transferase (HPRT) and glyceraldehyde-3-phosphate dehydrogenase (GAPDH) [58]. The best stability value was determined for the combination of ACTB and GAPDH genes (0.171). The relative PAC1 receptor transcript abundances were normalized relative to the geometric mean of ACTB and GAPDH mRNA abundances.

### 4.3. Western Blotting

The myometrial layers were homogenized on ice with a cold buffer (composition: 50 mmol/L Tris-HCl, pH 8.0; 150 mmol/L NaCl; 1% Triton X-100, 10 mg/mL aprotinin, 52 mmol/L leupeptin, 1 mmol/L pepstatin A, 1 mmol/l EDTA, 1 mol/L PMSF) and centrifuged (2500× *g* at 4 °C for 10 min). The supernatants were centrifuged (17,500× *g* at 4 °C for 1 h), and the collected supernatants were stored at −80 °C. The protein contents were determined using the Bradford method [59]. Protein extracts (20 μg) were dissolved in a sodium dodecyl sulfate (SDS) gel-loading buffer (composition: 50 mmol/L Tris-HCl, pH 6.8; 4% SDS, 20% glycerol and 2% β-mercaptoethanol), heated (at 95 °C for 4 min) and separated by 10% SDS-polyacrylamide gel electrophoresis. The separated proteins were then electroblotted onto a nitrocellulose membrane (0.22 μm) in transfer buffer (composition: 20 mmol/L Tris-HCl buffer, pH 8.2; 150 mmol/L glycine, 20% methanol, 0.05% SDS). To block the non-specific bindings, membranes were incubated with 5% fat-free dry milk in a TBS-T buffer at 21 °C for 1.5 h. They were then incubated at 4 °C for 18 h with primary PAC1 receptor polyclonal rabbit antibody (dilution: 1:1000, cat. no. CBS-PA207627, Cusabio Biotech Co.). Following rinsing in TBS-T buffer, the membranes were incubated at 21 °C, for 1 h with biotinylated goat anti-rabbit IgG (dilution: 1:3000, cat. no. PK-6101,Vectastain Elite ABC-HRP Kit, Vector Labs, Burlingame, CA, USA). Incubation (for 3–4 min) with a mixture of 3,3‘-diaminobenzidine tetrachloride (cat. no. D5637, Sigma Aldrich, St. Louis, MO, USA) and H2O2 in Tris-buffered saline (pH 7.2) was performed to visualize antibody binding. The negative control was prepared by removal of the primary antibody from the analysis. Proteins extracted from mice and porcine duodena were used as the positive controls. Images were gained and quantified using a Quantity-One system (VersaDoc 4000M imaging system, Bio-Rad Laboratories, Hercules, CA, USA). The band density was normalized in a ratio to relative GAPDH protein abundance.

### 4.4. Immunofluorescence

A cryostat (Reichert-Jung, Nußloch, Germany) was utilized to prepare sections (10 μm thickness) from uterine horns. The single-immunofluorescent method was used to estimate immunoreactivity to the PAC1 receptor [60]. Most importantly, the sections were incubated at 21 °C for 1 h with buffered blocking mixture (composition: 0.1 M PBS, 10% normal goat serum/MP Biomedicals, Solon, OH, USA/, 0.1% bovine serum albumin/Sigma-Aldrich, St. Louis, MO, USA/, 0.05% Thimerosal/Sigma-Aldrich, St. Louis, MO, USA/, 1% Triton X-100/Sigma-Aldrich, St. Louis, MO, USA/, 0.01% sodium azide) after drying at 21 °C for 30 min and rinsing (0.1M PBS, pH = 7.4, three times, each for 15 min). After washing (as described above), the sections were incubated at 21 °C for 18 h in a humid chamber with the same primary antibody against the PAC1 receptor (dilution: 1:200) as that used for Western blotting. The next day, after washing (as described above), the sections were incubated with biotinylated anti-rabbit IgG (dilution: 1:1000, cat. no. AP132B, Chemicon International, Temecula, CA, USA) at 21 °C for 1 h and then with carbocyanine 3 (CY3)-conjugated streptavidin (dilution: 1:9000, cat. no. 016160084, Jackson ImmunoResearch Labs, West Grove, PA, USA) at 21 °C for 1 h. The washed sections were then cover-slipped in carbonate-buffered glycerol (pH 8.6). The primary antibody was omitted to prepare the negative control. As a positive control, sections of porcine duodenum were used. Immunostained structures were analyzed and photographed using an Olympus BX51 microscope equipped with epifluorescence and the appropriate filter sets (Olympus, Consilio Sp. z o. o., Warsaw, Poland).

### 4.5. Contractility Measurement

The uterine contractile function was researched in accordance with the procedure outlined in [9]. Following rinsing in saline, strips of myometrium and endometrium/myometrium (3 × 5 mm) were mounted between two stainless steel hooks in an organ bath (10 mL capacity, Radnoti Unit Tissue Organ Bath System type 159920, Germany) under 5 mN tension. Krebs-Ringer solution (composition/mM/l/: NaCl, 120.3; KCl, 5.9; CaCl_2_, 2.5; MCl_2_, 1.2; NaHCO_3_, 15.5; glucose, 11.5; pH 7.4) was placed in a bath at 37 °C and was constantly saturated (mixture of 95% O_2_, 5% CO_2_).

The contractile function of the uteri was estimated based on two parameters: amplitude (the difference between the minimum and maximum values for a single contraction/mN/) and frequency (the number of peaks). The values of these parameters were registered by a force-displacement transducer, and the obtained data were analyzed using PowerChart software (Chart v5, scope v5, AD Instruments). The viability of strips and their usefulness for further research were determined using ACh (doses: 10^−7^, 10^−6^, 10^−5^ M, cat. no. A6625, Sigma, St. Louis, MO, USA), which was published previously [16]. Subsequently, PACAP (doses: 10^−8^, 10^−7^ M, cat. no. 1183, Tocris) was added, and the influence of its particular doses was registered for 10 min. The strips were then treated with PAC1 receptor antagonist (dose: 10^−6^ M, cat. no. 3236, Tocris) for 2 min, and PACAP (doses as given above) was then administered. The combined influence of the PAC1 receptor antagonist with PACAP was measured for 10 min. ACh (doses as given above) was utilized again at the end of the study to assess the strip viability. The doses of ACh were reported in [8,9], whereas PACAP and PAC1 receptor antagonist doses were estimated in the initial phase of research. For this purpose, strips from healthy pig uteri were treated with PACAP only (doses: 10^−9^, 10^−8^ and 10^−7^ M) and in combination with PAC1 receptor antagonist (doses: 10^−8^, 10^−7^ and 10^−6^ M). It was noted that PACAP most effectively changed the contractility at doses of 10^−8^ and 10^−7^ M, and this antagonist at a dose of 10^−6^ M statistically significantly modified the contractile function induced by PACAP (data not shown).

### 4.6. Statistical Analyses

Mean (±SEM) relative PAC1 receptor mRNA transcript and protein abundances were counted for each group. In the contractile studies, only the results for which the variability (the parameters obtained in response to ACh between the beginning and the end of the investigation) was below 20% were statistically analyzed. Mean (±SEM) values of amplitude and frequency were counted for a particular group before the addition of substances (pre-treatment period) were accepted as 100%. The actions of substances were expressed as the percentage (mean ± SEM) change from values measured before their use. The uterine contractility analyses included comparisons between (1) mean values before and after each treatment (PACAP alone, PACAP and PAC1 receptor antagonist) in each group, (2) mean values between groups in response to the same treatment (PACAP alone, PACAP and PAC1 receptor antagonist) and (3) mean values between treatments (PACAP and PAC1 receptor antagonist versus PACAP alone) for each group/PACAP dose. The statistical significance of the obtained data was assessed by a Bonferroni test (ANOVA, InStat Graph Pad, San Diego, CA). Statistically significant differences were indicated by: * *p* <0.05, ** *p*<0.01, *** *p* <0.001. All obtained data were normally distributed. No statistical power calculation was conducted prior to the research, and the establishment of the size of experimental groups was based on previous studies, in which samples of five animals (in the case of pigs during uterine investigations) were commonly accepted.

## 5. Conclusions

In the current study, we found that severe acute endometritis decreases the relative abundance of PAC1 receptor protein in the pig myometrium. In the context of endometritis, PACAP (through the PAC1 receptor) enhances the contractile amplitude in the myometrium. These results suggest a possible control function for PACAP in uterine contractility during a spontaneous inflammatory process. The obtained data may be the basis for future research aimed at studying the mechanisms of alterations in PAC1 receptor content and, consequently, the importance of this receptor in the prevention and treatment of myometrial contractility disorders during uterine inflammation.

## Figures and Tables

**Table 1:** TaqMan assays used in the research.

Symbol	Name	Assay No.
*PAC1R*	*adenylate cyclase-activating polypeptide (pituitary) receptor type I*	*Ss04248302_m1*
*GAPDH*	*glyceraldehyde-3-phosphate dehydrogenase*	*Ss03375435_u1*
*ACTB*	*β-actin*	*Ss03376081_u1*
*HPRT*	*hypoxanthine guanine phosphoribosyl transferase*	*Ss03388274_m1*

## Data Availability

All relevant data are contained within the manuscript.

## Supplementary Materials

The following supporting information can be downloaded at: https://www.mdpi.com/article/10.3390/ijms23105467/s1.
