# Supplementary material for: Investigation of the Role of Pituitary Adenylate Cyclase-Activating Peptide (PACAP) and Its Type 1 (PAC1) Receptor in Uterine Contractility during Endometritis in Pigs
Source: Int J Mol Sci. 2022 May 13;23(10):5467. doi: 10.3390/ijms23105467 (PMC9146118; doi:10.3390/ijms23105467)
Supplement: Supplementary file 1 [file ijms-23-05467-s001.zip › ijms-1686572-supplementary.pdf]

Supplementary material

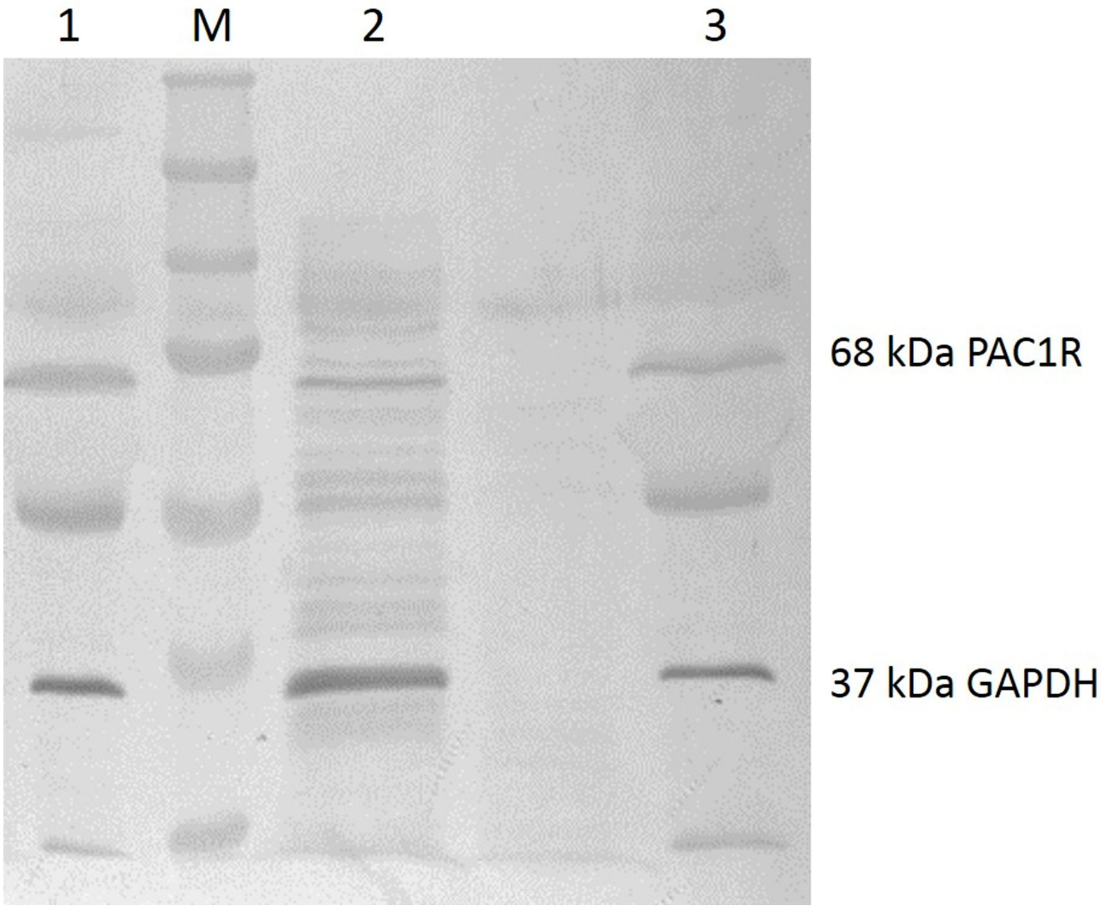

Supplementary Figure S1. Western blot analysis of pituitary adenylate cyclase-activating peptide receptor (PAC1R) protein in porcine myometrium and relevant positive control. For PAC1R antibody bands at 68 kDa are visible in porcine myometrium (line 1) and mouse (line 2) and porcine (line 3) duodenum. M - marker.

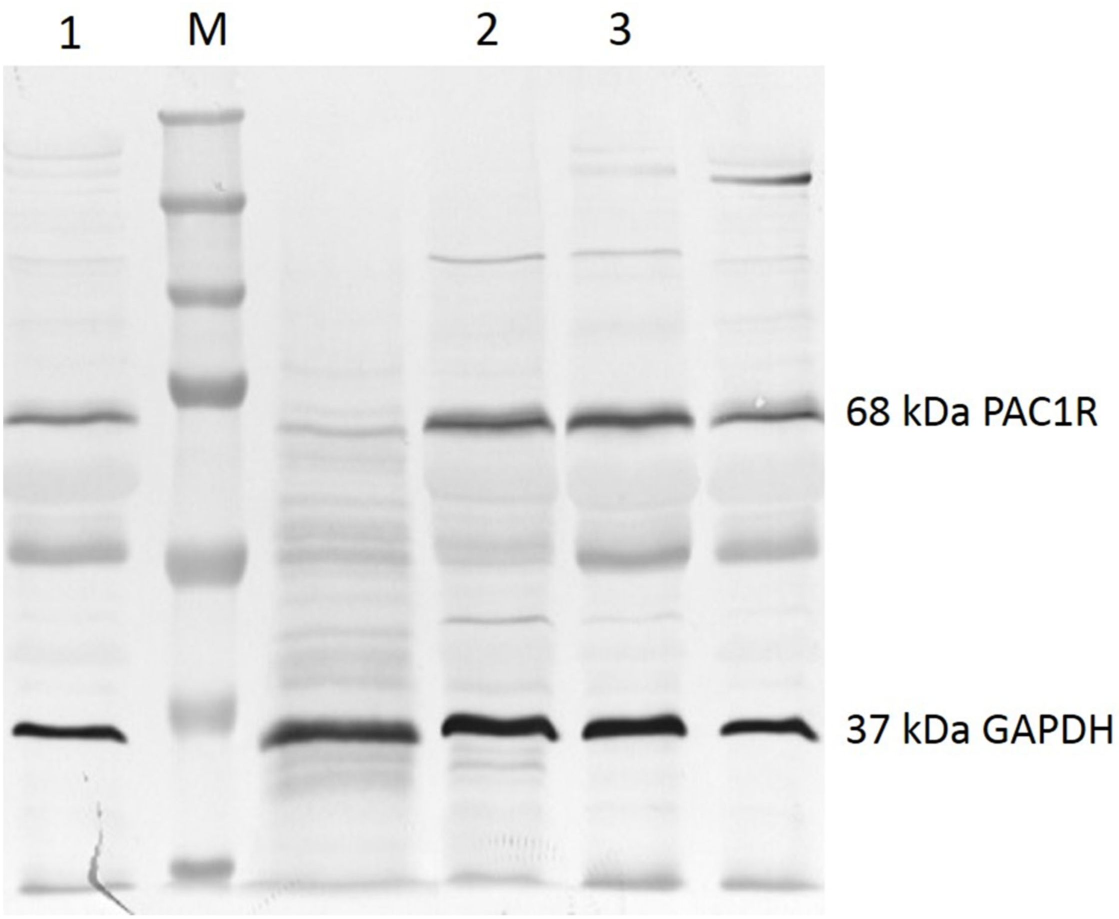

Supplementary Figure S2. Blot shows representative bands of pituitary adenylate cyclase-activating peptide receptor (PAC1R) protein expression in the myometrial layer of gilts from the *E. coli* (*E. coli*; line 1), control (CON; line 2) and saline (SAL; line 3) groups, estimated by Western blot analysis. The density of bands was normalized in relation to glyceraldehyde-3-phosphate dehydrogenase (GAPDH). M - marker.

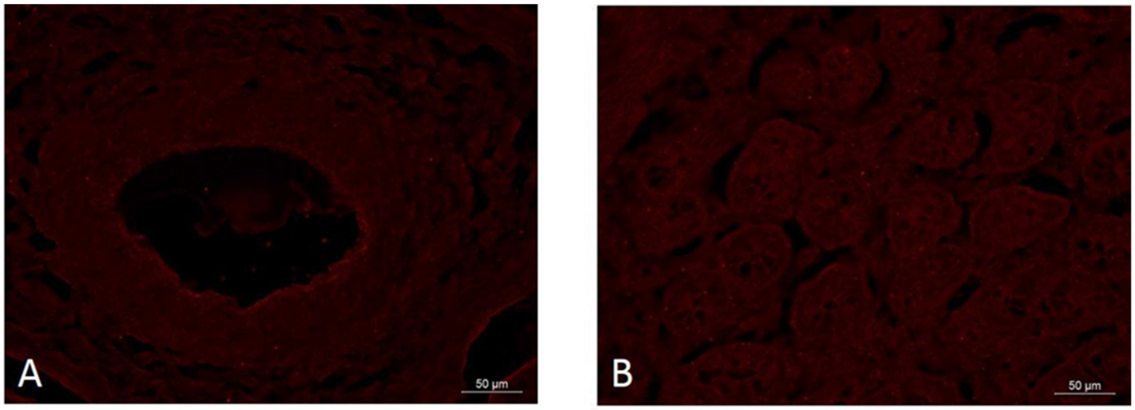

Supplementary Figure S3. The immunoreaction of pituitary adenylate cyclase-activating peptide receptor (PAC1R) in porcine myometrium and relevant positive control. Pictures show the presence of PAC1R in porcine myometrium (A) and duodenum (B).
